# Oral Health Disparities among Illicit Drug Users in the US: Secondary Analysis using Data from NHANES 2017–2018

**DOI:** 10.3290/j.ohpd.c_2420

**Published:** 2026-01-22

**Authors:** Nada Farsi, Heba Ashi, Abdulraheem Alwafi, Layla Abuljadayel, Meyassara Samman, Mohamed Bamashmous, Dalia Meisha, Dania Sabbahi

**Affiliations:** a Nada Farsi Associate Professor, Department of Dental Public Health, Faculty of Dentistry, King Abdulaziz University, Jeddah, Saudi Arabia. Analyzed the data and results, co-wrote the manuscript.; b Heba Ashi Associate Professor, Department of Dental Public Health, Faculty of Dentistry, King Abdulaziz University, Jeddah, Saudi Arabia. Analyzed the data and results, co-wrote the manuscript.; c Abdulraheem Alwafi Assistant Professor, Department of Dental Public Health, Faculty of Dentistry, King Abdulaziz University, Jeddah, Saudi Arabia. Analyzed the data and results, co-wrote the manuscript.; d Layla Abuljadayel Assistant Professor, Department of Dental Public Health, Faculty of Dentistry, King Abdulaziz University, Jeddah, Saudi Arabia. Conceived study idea, co-wrote the manuscript.; e Meyassara Samman Associate Professor, Department of Dental Public Health, Faculty of Dentistry, King Abdulaziz University, Jeddah, Saudi Arabia. Conceived study idea, co-wrote the manuscript.; f Mohamed Bamashmous Associate Professor, Department of Dental Public Health, Faculty of Dentistry, King Abdulaziz University, Jeddah, Saudi Arabia. Wrote the manuscript.; g Dalia Meisha Associate Professor, Department of Dental Public Health, Faculty of Dentistry, King Abdulaziz University, Jeddah, Saudi Arabia. Conceptualized the idea, interpreted the findings, drafted selected sections, and critically revised the manuscript.; h Dania Sabbahi Associate Professor, Department of Dental Public Health, Faculty of Dentistry, King Abdulaziz University, Jeddah, Saudi Arabia. Wrote the manuscript.

**Keywords:** Decayed, Missing and Filled Teeth Index, illicit drug usage, marijuana, substance abuse.

## Abstract

**Purpose:**

This study used data from the National Health and Nutrition Examination Survey (NHANES) to examine associations between the use of four illicit drugs (cocaine, heroin, marijuana, and methamphetamine) and the dental caries experience among adults in the US using the Decayed, Missing and Filled Teeth (DMFT) index.

**Materials and Methods:**

Data from NHANES 2017–2018, a cross-sectional study that included adults aged 18–69 years, was used in this study. A self-reported questionnaire was used to collect data related to drug use, and a calibrated dental examination was performed to diagnose caries. The associations among the illicit drug use, DMFT score, and score of each component were analyzed using zero-inflated negative binomial regression.

**Results:**

Marijuana was the most commonly used drug among the participants (60.9%). In adjusted zero-inflated negative binomial models, current heroin was associated with fewer missing teeth (IRR = 0.50, 95% CI: 0.25 – 0.82) and former heroin use with fewer decayed teeth (IR R= 0.61, 95% CI: 0.41 – 0.97). The associations for marijuana and cocaine were not statistically significant after adjustment.

**Conclusion:**

Illicit drug use shows heterogenous associations with the development of caries; however, the impact differs according to the type of drug. The findings of this study emphasize the requirement for comprehensive dental management and integration of substance-abuse screening within oral healthcare services.

The findings of the 2021 National Survey on Drug Use and Health (NSDUH) demonstrate that the prevalence of substance use in the United States is a matter of great concern. The NSDUH revealed that 61.2 million individuals aged ≥12 years, equating to 21.9% of the total population, reported using illicit substances. Marijuana was reported as the most frequently used illicit substance, followed by cocaine, lysergic acid diethylamide (LSD), ecstasy, methamphetamine, and heroin. Furthermore, 40 million individuals (14.5%) aged ≥12 years had used an illicit drug within the preceding 30-day period. Among these individuals engaging in illicit substance use, 24 million individuals aged ≥12 years met the criteria for drug use disorder.^41^

Illicit drug use has wide-ranging consequences on physical, mental, and social well-being. It is associated with serious health risks, including cardiovascular, hepatic, and respiratory complications, as well as a heightened likelihood of infectious diseases from injectable substances.^8,23^ Mental health effects such as depression, anxiety, and fatal overdoses are common, particularly among those using narcotics or mixed drugs.^4,19^ Drug misuse can also strain interpersonal relationships, leading to neglect, aggression, and emotional distress,^13^ while social isolation and stigma may discourage individuals from seeking help.^10^ Beyond personal health, drug abuse reduces workplace productivity, increases healthcare and law enforcement costs, and contributes to financial instability.^17,38^ Importantly, illicit drug use is strongly linked to oral health problems, including widespread caries, periodontal disease, mucosal dysplasia, bruxism, attrition, and tooth loss.^34^

The etiology of caries involves a dynamic interplay between several critical factors. The core process involves the interaction of a susceptible tooth surface with cariogenic bacteria present in the dental biofilm, such as Streptococcus mutans and Lactobacillus, and the cariogenic effects of fermentable carbohydrates, particularly sugars and refined starch.^27^ Several modifying factors, such as dietary habits, salivary flow and composition, and fluoride exposure, play crucial roles in the development of caries. Moreover, factors such as oral hygiene practices, socioeconomic status, and individual genetics also exhibit significant effects on the risk and progression of caries.^32^

Illicit drug use—defined here as marijuana, cocaine, heroin, or methamphetamine, with use status (never, former, or current) based on self-report—has wide-ranging consequences on physical, mental, and social well-being. Drugs, such as cannabis, methamphetamine, cocaine, and heroin, can cause xerostomia and decrease the buffering capacity of saliva.^2,7,28,30^ Reduction in salivary flow can disrupt the oral environment, thereby increasing the risk of developing caries, periodontal disease, and oral infections. Increased sugar intake among illicit drug users^29,37^ increases the availability of substrates for the growth of cariogenic bacteria and intensity of acid attacks, thereby resulting in the development of caries. Furthermore, the oral administration of drugs, such as methamphetamine and cocaine, can create a more acidic environment owing to their acidic nature.^39^ The effects of these factors on the development of caries are intensified in individuals with poor oral hygiene.

Two recent studies analyzed data from the National Health and Nutrition Examination Survey (NHANES; 2009–2014) to evaluate the oral health status of drug users in the United States. The first study assessed the oral health status of known methamphetamine users and reported that the prevalence of untreated caries, periodontitis, and severe periodontitis among methamphetamine users was higher than that in non-users (36.6%, 54.8%, and 12.2%, respectively).^20^ The second study assessed the oral health status of known cocaine users and reported that the prevalence of untreated caries, periodontitis, and severe periodontitis among cocaine users was higher than that in non-users (29.8%, 47.3%, and 11.4%, respectively).^3^ However, these studies only focused on only one drug and assessed the presence of caries without quantifying the severity of the disease. Therefore, analyzing NHANES data is necessary to assess the impact of multiple drugs such as cocaine, methamphetamine, and marijuana, on the severity of caries.

No previous study has explored the association between drug use and oral health outcomes in the NHANES cohort (2017–2018). Therefore, this study used data from the NHANES 2017–2018 to evaluate the association between the use of four illicit drugs (cocaine, heroin, marijuana, and methamphetamine) and severity and experience of caries in the US using the Decayed, Missing and Filled Teeth (DMFT) index.

## MATERIALS AND METHODS

The present study utilized publicly available data from the National Health and Nutrition Examination Survey (NHANES) 2017-2018. NHANES data is a valuable resource freely accessible for download from the Centers for Disease Control and Prevention (CDC) website: https://wwwn.cdc.gov/nchs/nhanes/continuousnhanes/default.aspx?BeginYear=2017.

### Study Design and Population 

Data from the NHANES, specifically the data collected between 2017 and 2018, were used in this study. The target population comprised adults aged 18–69 years. NHANES uses a complex, multistage stratified probability sample of the civilian, non-institutionalized U.S. population. Primary sampling units (PSUs; counties or groups of counties) are selected within strata, followed by selection of area segments (census tracts/blocks), households, and finally individuals, with over-sampling of key subgroups. The NHANES aimed to assess the health and nutritional status of adults and children in the United States. A combination of interviews and physical examinations were performed to obtain a broad range of data on various health parameters, such as the prevalence of chronic disease, nutritional status, and risk factors for diseases.

### Substance Use Assessment 

Variables reflecting lifetime use of illicit drug use were created for each drug, and the participants were divided into different categories based on the self-reported use of cocaine, marijuana, heroin, and methamphetamine. The variable created for quantity and units of last drug use indicated the last instance the drug was used (in years). A new categorical variable indicating lifetime drug use was also created. The users were classified into three categories: never users, former users (drugs used before but not in the last year), or current users (drugs used within the last year), for each substance.

### Oral Health Assessment

NHANES-calibrated dentists performed dental examinations to assess the prevalence of untreated caries and missing and filled teeth. The DMFT scores were calculated based on the findings of the examination. The scores for the individual components, i.e., the decayed, missing, and filled teeth, were generated initially. The scores were derived based on specific conditions identified in the questionnaire. The total DMFT score was defined as the sum of the scores for decayed, missing, and filled teeth.

### Demographic and Confounding Variables

Variables, including age, sex, race, socioeconomic status, educational level, and rehabilitation history, were collected and included in the analysis to control for potential confounders.

### Ethical Consideration

The Institutional Review Board of the National Center for Health Statistics (NCHS) approved the protocols for the NHANES. Written informed consent was obtained from all participants. The Research Ethics Committee of King Abdulaziz University, Faculty of Dentistry, Jeddah, Saudi Arabia, approved the protocol for the secondary data analysis.

### Statistical Analysis

The demographic characteristics and prevalence of illicit drug use among participants are presented as unweighted counts and survey-weighted percentages.

The prevalence of DMFT and its components was examined based on the status of illicit drug use. DMFT and its components were initially categorized into binary variables: 0 indicated the absence of the condition, whereas 1 indicated the presence of at least one tooth affected by the condition. Weighted percentages were calculated for each group subsequently. The association between drug use and the presence or absence of DMFT and its components for each substance were assessed using the chi-squared test. The means and standard errors of the DMFT scores and the components of DMFT were calculated across different categories of illicit drug use (never, current, and former users).

The relationship between illicit drug use and DMFT and the scores of each of its components were assessed separately using zero-inflated negative binomial regression models. Univariate regression was performed for each class of drug (marijuana, cocaine, heroin, and methamphetamine). Multivariate regression analyses were performed subsequently after adjusting for other drugs and the following variables: age, sex, race/ethnicity, education level, family income:poverty ratio, and rehabilitation history. A zero-inflated negative binomial (ZINB) model was selected because the count outcomes (DMFT, D, M, and F) were overdispersed. To confirm model appropriateness, we compared ZINB and NB models using AIC and BIC, and the ZINB model demonstrated a better fit. The covariates in the model included age, sex, race/ethnicity, education, income, rehabilitation history, and illicit drug use.

All analyses incorporated the NHANES complex survey design. MEC examination weights were applied, and the analyses incorporated the specified strata and primary sampling units in accordance with NHANES analytic guidelines. Standard errors were computed using Taylor-series linearization commands.

All statistical analyses were two-tailed and performed using Stata version 12.1 (StataCorp; College Station, TX, USA). A p-value of ≤0.05 was considered statistically significant.

## RESULTS

The use of marijuana (or hashish) was reported by 62% of participants. The highest prevalence of marijuana use (66.8%) was observed among participants aged 18-29 years. The use of cocaine, methamphetamine, and heroin was reported by 17.3%, 8.5%, and 2.8% of participants, respectively. The prevalence of illicit drug use among men was higher than among women. The prevalence of illicit drug use among non-Hispanic White individuals was higher than that among individuals of other ethnicities. Only 9.4% of the participants had entered a rehabilitation program (Table 1).

**Table 1:** Demographic characteristics and illicit drug use among participants aged 18–69 y in the NHANES 2017–2018

Characteristic	Category	n (unweighted)	Overall % (SE)	Marijuana Ever % (SE)	Cocaine Ever % (SE)	Heroin Ever % (SE)	Methamphetamine Ever % (SE)
Age (years)	18–29	1,115	24.8 (1.3)	66.8 (1.5)	12.9 (1.5)	2.8 (0.9)	4.5 (1.3)
	30–44	1,267	28.9 (1.0)	62.7 (2.5)	18.0 (1.8)	1.5 (0.5)	9.9 (1.3)
	45–59	1,324	30.3 (1.1)	54.4 (2.4)	20.2 (1.9)	4.0 (0.8)	11.0 (1.5)
	60–69	1,019	16.1 (1.3)	N/A	N/A	N/A	N/A
Sex	Male	2,312	49.0 (0.9)	65.0 (2.0)	21.2 (1.6)	4.1 (0.8)	11.0 (1.6)
	Female	2,498	51.0 (0.9)	56.8 (1.7)	13.4 (1.1)	1.5 (0.4)	6.0 (0.8)
Race/Ethnicity	Mexican American	722	9.9 (1.8)	48.9 (2.5)	17.9 (2.9)	2.2 (0.8)	9.8 (1.4)
	Other Hispanic	479	7.5 (0.8)	40.6 (3.3)	12.8 (2.2)	2.4 (0.7)	5.2 (1.6)
	Non-Hispanic White	1,427	59.7 (2.5)	66.8 (2.5)	19.9 (1.7)	3.4 (0.7)	10.3 (1.4)
	Non-Hispanic Black	1,161	11.9 (1.6)	65.0 (2.5)	11.1 (1.3)	1.7 (0.6)	2.3 (0.9)
	Other/Multi-racial	1,021	11.1 (1.3)	50.8 (3.7)	11.2 (1.8)	1.2 (0.3)	5.5 (1.3)
Education	<HS	931	11.2 (0.9)	48.9 (3.4)	20.7 (1.4)	5.5 (1.1)	11.1 (1.6)
	HS or GED	1,211	28.1 (1.7)	62.5 (1.8)	19.5 (2.1)	3.8 (1.3)	10.4 (2.0)
	>HS	2,661	60.7 (2.2)	62.1 (2.0)	15.7 (1.3)	1.8 (0.4)	7.2 (0.8)
Income-to-poverty	Below Poverty Line	871	14.4 (1.0)	60.5 (2.8)	23.4 (2.4)	4.6 (1.2)	12.5 (2.0)
	Low to Moderate Income	1,762	34.8 (2.0)	62.9 (1.6)	19.4 (1.8)	3.3 (1.1)	10.9 (1.8)
	Middle to High Income	1,491	50.9 (2.0)	63.0 (2.7)	15.6 (1.5)	2.3 (0.5)	6.3 (0.8)
Rehab history	Yes	186	9.4 (1.0)	93.9 (2.7)	79.8 (3.5)	31.1 (4.0)	43.4 (5.8)
	No	1,579	90.6 (1.0)	99.1 (0.2)	22.7 (1.5)	1.8 (0.5)	11.0 (1.7)
Counts are unweighted; SEs are survey-weighted using NHANES MEC examination weights and account for the multistage, stratified design with strata and primary sampling units using Taylor-series linearization. NHANES, National Health and Nutrition Examination Survey; HS, high school; GED, General Educational Development.

Table 2 presents the prevalence of DMFT and its components and their associations with illicit drug use. Approximately 84.9%, 81.3%, and 87.7% of never, current, and former users of marijuana, respectively, had a DMFT score of ≥1 (P=0.036). The prevalence of decayed teeth (D) among current and former users of all classes of drugs was noticeably greater than that among never users, with the exception of marijuana users. The number of individuals with at least one missing tooth was higher among users of heroin and methamphetamine, both in the past and current categories. However, the prevalence of missing teeth among never users of marijuana was higher than that among current and former users of marijuana. Similarly, the prevalence of missing teeth among former users of cocaine was also higher than that among current or never users of cocaine. The prevalence of filled teeth (F) among current users of marijuana was lower than that among never and former users of marijuana (p = 0.025).

**Table 2:** Prevalence of DMFT and its indicators according to illicit drug use among participants aged 18–69 years in the NHANES 2017–2018

Outcome	Status of usage	Marijuana or Hashish %	N	p-value*	Cocaine %	N	p-value*	Heroin %	N	p-value*	Metham- phetamine %	N	p-value*
Any DMFT	Never used	84.9	1,461	0.036**	86.7	3,503	0.107	86.9	4,036	0.163	86.9	3,858	0.732
	Current user	81.3	911		80.4	123		96.8	22		89.8	66	
	Former user	87.7	827		90.7	516		91.2	85		88.7	219	
Any D	Never used	17.3	1,461	0.122	17.4	3,503	0.015**	18.0	4,036	0.004**	17.2	3,858	0.001**
	Current user	22.8	911		29.6	123		55.1	22		48.4	66	
	Former user	17.3	827		22.1	516		31.3	85		28.5	219	
Any M	Never used	41.5	1,461	0.081**	42.7	3,503	<0.001**	43.5	4,036	0.049**	42.8	3,858	<0.001**
	Current user	33.1	911		34.4	123		60.0	22		72.6	66	
	Former user	37.8	827		55.1	516		66.5	85		55.2	219	
Any F	Never used	76.7	1,461	0.025**	77.3	3,503	0.093	76.7	4,036	0.788	76.9	3,858	0.226
	Current user	70.1	911		65.9	123		74.7	22		64.0	66	
	Former user	78.9	827		74.7	516		72.2	85		74.4	219	
*Chi-squared test. **Statistically significant at p < 0.05. Survey-weighted estimates with design-adjusted SEs using NHANES MEC examination weights; analyses account for the multistage, stratified design with strata and primary sampling units via Taylor-series linearization. NHANES, National Health and Nutrition Examination Survey; DMFT, Decayed, Missing and Filled Teeth; D, decayed; M, missing; F, filled.

Table 3 illustrates the mean scores and standard errors of DMFT and its components among participants with varying histories of drug use. The DMFT scores ranged from as low as 6.2 (0.8) among current users of cocaine to as high as 11.0 (1.4) among former users of heroin. The decayed teeth score was lowest for never users of marijuana (0.4 [0.1]), whereas it was the highest for current users of heroin (1.9 [0.4]). The missing teeth scores were 1.7 (0.2) and 5.7 (1.0) for current users of marijuana and former users of heroin, respectively. The filled teeth scores were 3.4 (0.4) and 5.7 (0.5) for current users of methamphetamine and former users of marijuana, respectively.

**Table 3:** Mean DMFT and its indicators according to the drug use status among participants aged 18–69 years in the NHANES 2017–2018

Outcome	Status of usage	Marijuana or Hashish Mean (SE)	n	Cocaine Mean (SE)	n	Heroin Mean (SE)	n	Methamphetamine Mean (SE)	n
DMFT	Never used	7.7 (0.2)	1,461	8.6 (0.3)	3,503	8.8 (0.3)	4,036	8.7 (0.3)	3,858
	Current user	6.5 (0.2)	911	6.2 (0.8)	123	7.1 (1.4)	22	10.6 (1.1)	66
	Former user	8.3 (0.5)	827	10.4 (0.5)	516	11.0 (1.4)	85	9.8 (0.6)	219
D	Never used	0.4 (0.1)	1,461	0.5 (0.04)	3,503	0.5 (0.04)	4,036	0.5 (0.04)	3,858
	Current user	0.7 (0.1)	911	0.9 (0.2)	123	1.9 (0.4)	22	1.6 (0.4)	66
	Former user	0.5 (0.1)	827	0.7 (0.1)	516	1.0 (0.4)	85	0.9 (0.2)	219
M	Never used	2.0 (0.2)	1,461	2.6 (0.2)	3,503	2.8 (0.2)	4,036	2.7 (0.2)	3,858
	Current user	1.7 (0.2)	911	1.8 (0.3)	123	1.7 (0.5)	22	5.7 (1.0)	66
	Former user	2.0 (0.2)	827	4.5 (0.5)	516	5.7 (1.3)	85	3.9 (0.6)	219
F	Never used	5.3 (0.2)	1,461	5.6 (0.2)	3,503	5.5 (0.2)	4,036	5.5 (0.2)	3,858
	Current user	4.0 (0.2)	911	3.5 (0.4)	123	3.6 (0.9)	22	3.4 (0.4)	66
	Former user	5.7 (0.4)	827	5.1 (0.5)	516	4.4 (0.8)	85	5.0 (0.5)	219
Survey-weighted estimates with design-adjusted SEs using NHANES MEC examination weights; analyses account for the multistage, stratified design with strata and primary sampling units via Taylor-series linearization. NHANES, National Health and Nutrition Examination Survey; DMFT, Decayed, Missing and Filled Teeth; D, decayed; M, missing; F, filled.

Table 4 presents the results of univariate and multivariate regression analyses performed to assess the associations between drug use and DMFT, as well as its components. Univariate analyses revealed that current use of marijuana was correlated with a decrease in the DMFT score, whereas former use of cocaine was correlated with an increase in the DMFT score, compared with that in never users. Current use of marijuana was correlated with an increase in the decayed teeth scores. Former use of cocaine use was correlated with an increase in the missing teeth score, whereas current use of heroin was correlated with a decrease in the missing teeth score. Current use of all classes of assessed drugs was correlated with a lower filled teeth score. Multivariate regression analysis revealed an overall decrease in these associations; however, former and current use of heroin was correlated with a decrease in the decayed teeth and missing teeth scores, respectively. Furthermore, former use of methamphetamine was associated with a decrease in the missing teeth score.

**Table 4:** Univariate and multivariate regression models for analyzing the correlation between illicit drug use and DMFT and its indicators among participants aged 18–69 years in the NHANES 2017–2018

Variable	DMFT IRR (95% CI)	Decayed teeth IRR (95% CI)	Missing teeth IRR (95% CI)	Filled teeth IRR (95% CI)
Univariate				
Marijuana Use				
Never	1.0	1.0	1.0	1.0
Current	0.90 (0.82, 0.91)	1.35 (1.11, 1.65)	1.11 (0.90, 1.35)	0.82 (0.74, 0.90)
Former	1.04 (0.90, 1.11)	1.35 (0.96, 1.82)	1.11 (0.90, 1.49)	1.05 (0.99, 1.11)
Cocaine Use				
Never	1.0	1.0	1.0	1.0
Current	0.74 (0.61, 1.01)	1.11 (0.90, 1.35)	0.90 (0.67, 1.22)	0.74 (0.55, 0.97)
Former	1.11 (1.01, 1.35)	1.22 (0.82, 1.49)	1.35 (1.11, 1.65)	0.95 (0.82, 1.08)
Heroin Use				
Never	1.0	1.0	1.0	1.0
Current	0.74 (0.50, 1.11)	1.22 (0.98, 1.65)	0.50 (0.27, 0.82)	0.67 (0.50, 0.90)
Former	1.22 (0.90, 1.65)	1.11 (0.55, 2.46)	1.35 (0.82, 2.22)	0.82 (0.61, 1.11)
Methamphetamine Use				
Never	1.0	1.0	1.0	1.0
Current	1.22 (1.00, 1.49)	1.22 (0.90, 1.65)	1.22 (0.82, 1.82)	0.74 (0.61, 0.90)
Former	1.11 (0.97, 1.22)	1.11 (0.74, 1.65)	1.11 (0.82, 1.49)	0.90 (0.82, 1.11)
Multivariate*				
Marijuana Use				
Never	1.0	1.0	1.0	1.0
Current	0.90 (0.61, 1.35)	0.90 (0.45, 2.01)	1.22 (0.67, 2.22)	0.90 (0.55, 1.49)
Former	0.90 (0.61, 1.49)	0.95 (0.45, 2.01)	1.22 (0.61, 2.22)	0.97 (0.61, 1.65)
Cocaine Use				
Never	1.0	1.0	1.0	1.0
Current	0.90 (0.67, 1.22)	1.04 (0.67, 1.65)	0.90 (0.61, 1.49)	0.90 (0.74, 1.22)
Former	1.05 (0.90, 1.22)	1.11 (0.82, 1.49)	1.49 (0.90, 2.46)	0.96 (0.82, 1.11)
Heroin Use				
Never	1.0	1.0	1.0	1.0
Current	0.90 (0.61, 1.35)	0.82 (0.45, 1.65)	0.50 (0.25, 0.82)	1.01 (0.74, 1.35)
Former	0.90 (0.67, 1.35)	0.61 (0.41, 0.96)	0.82 (0.45, 1.49)	0.90 (0.61, 1.22)
Methamphetamine Use				
Never	1.0	1.0	1.0	1.0
Current	1.22 (1.00, 1.49)	1.03 (0.67, 1.65)	1.22 (0.74, 2.01)	0.90 (0.74, 1.35)
Former	1.00 (0.90, 1.11)	0.74 (0.50, 1.22)	0.61 (0.41, 0.97)	1.04 (0.90, 1.22)
Incidence rate ratios (95% CI) from zero-inflated negative binomial models, survey-weighted with NHANES MEC examination weights and design-adjusted for NHANES strata and primary sampling units. Sample sizes (n) for each drug use category are provided in Tables 1–3. All models were adjusted for age, sex, race/ethnicity, education level, ratio of family income to poverty, history of entering a rehabilitation program, and other drugs use. Collinearity among drug-use variables was evaluated using variance inflation factors (VIF) (range 1.14–1.80), indicating no multicollinearity concerns. NHANES, National Health and Nutrition Examination Survey; DMFT, Decayed, Missing and Filled Teeth; CI, confidence interval.

## DISCUSSION 

The findings of this analysis suggest that illicit drug use is linked to heterogeneous patterns in caries experience, with the magnitude and direction of associations varying by drug type. Drug use was associated with differences in DMFT components. The induction of dry mouth or xerostomia by illicit substances, such as crack cocaine, is a significant contributor to this risk.^2,30^ Saliva plays a crucial role in neutralizing acids, remineralizing enamel, and removing food particles. However, this protective effect is diminished when the salivary flow is reduced,^26,44^ thereby increasing the susceptibility to caries. Drug use is often correlated with poor oral hygiene, as these individuals may not brush or floss.^21,43^ Poor oral hygiene, combined with lifestyle factors, such as altered dietary habits, can lead to the accumulation of plaque, fostering an environment conducive to the growth of cariogenic bacteria.^14^ Furthermore, the use of some drugs induces bruxism or tooth grinding, which results in tooth wear and fractures, in addition to increasing the vulnerability to caries.^40^

Correlations among drug abuse, neglect, and avoidance of dental care were observed despite the evident need for dental care.^10^ This avoidance persists even in the presence of pain or discomfort. Individuals with substance use disorders are often unable to access dental treatment. The reluctance to seek dental treatment is multifaceted; however, heightened dental anxiety among illicit drug users is a major contributing factor.^32^ Fear of dental procedures, coupled with perceived judgment from dental professionals, deters individuals with substance use disorders from seeking treatment. Moreover, substance users frequently experience psychiatric difficulties, which compound the challenges associated with maintaining oral health.^33^ Mental health issues may further hinder the prioritization and management of dental concerns. The interplay between substance use, mental health disorders, and dental avoidance creates a complex scenario that can only be mitigated via a holistic and interdisciplinary approach.^19^

Previous studies investigating the relationship between cocaine use, especially crack cocaine, and caries revealed that the prevalence of caries among cocaine users was consistently higher. The prevalence and severity of caries among users of crack cocaine were statistically significantly higher than those among non-users, as indicated by the higher DMFT scores.^1,2,3,12^ An increase in the DMFT scores and prevalence of missing teeth was also observed among former users of cocaine in the present study. In addition, a decrease in the prevalence of filled teeth was observed among current users of cocaine.

An increase in the prevalence of caries and a decrease in the prevalence of filled teeth were observed among current users of marijuana in the present study. The DMFT scores of current users of marijuana were lower than those of former users of cocaine. Several studies have evaluated the effects of the use of cannabis on the severity of caries; however, the findings of these studies were inconsistent. A study conducted in the US reported a statistically significant increase in the severity of caries among adolescents who used tobacco and marijuana, suggesting a notable negative impact on dental health.^15^

In contrast, a study conducted in Switzerland did not report an increase in tooth decay among cannabis users.^31^ A recent literature review highlighted the sparse and inconsistent evidence directly linking cannabis smoking to caries; however, potential underlying risk factors were reported in this study.^22^ Thus, although the use of cannabis may contribute to an increased risk of developing caries, this association is likely influenced by lifestyle and oral hygiene practices. Nevertheless, further studies must be conducted to clarify this relationship.

The present study revealed a lower prevalence of decayed teeth and missing and filled teeth among former and current users of heroin, respectively. However, several previous studies have collectively indicated a strong association between the use of heroin and poor oral health, particularly in terms of caries. A study conducted in Iran revealed that the prevalence of missing teeth was higher among individuals who are dependent on opiates, especially those using heroin. Moreover, a statistically significant proportion of the DMFT scores were related to missing teeth, indicating a history of caries.^34^ A study from China revealed a high prevalence of decayed or filled teeth and roots, along with notable rates of missing teeth, among former users of heroin. The findings of this study suggest that the oral health of these individuals was poorer than that of the general population.^24^ Another study from China reported a high prevalence of caries and DMFT scores among users of heroin. The majority of the DMFT score was related to decay; only a small portion was related to treatment.^16^ Thus, heroin use is closely linked to an increased risk of caries and the overall deterioration of oral health. These findings collectively underscore the requirement for targeted dental care interventions in this population.

The present study revealed a lower prevalence of missing teeth among former users of methamphetamine and a lower prevalence of filled teeth among current users of methamphetamine, which is in contrast with the findings of other studies that indicated the detrimental effect of methamphetamine on oral health, particularly in terms of increased caries and missing and filled teeth.^5,20,35,36,42^

The association between drug use and the prevalence of DMFT and its components differed based on the class of drug in the present study. No study has identified the reason for this difference; however, one possible explanation could be that the difference in chemical composition has a different magnitude of impact among users of these drugs.

The findings of the present study are consistent with those of a previous study, which reported that the association between methamphetamine use and the presence of carious and missing teeth was influenced by race/ethnicity.^5^

The present study used high-response-rate data from NHANES, a nationally representative survey. To the best of our knowledge, this is the first study to elucidate the association between the use of different illicit drugs and the severity of caries among adults in the US using a detailed component of the DMFT. Previous studies evaluated the presence or absence of caries; however, the disease severity was not evaluated. Moreover, these studies focused on a single drug and examined the presence of caries without gauging disease severity. In contrast, the present study investigated the link between the use of four illicit drugs (cocaine, heroin, marijuana, and methamphetamine) and the severity of caries using the DMFT index via zero-inflated negative binomial regression, which is strength of the statistical power of the study. Thus, the present study offers enhanced predictions and estimates by efficiently managing excess zeros, distinguishing between zero types, and addressing data overdispersion.

### Limitations

The present study offers valuable insights, but some limitations are important to consider. First, the cross-sectional nature of NHANES hindered the establishment of cause-and-effect relationships. Furthermore, the data were collected via a self-reported questionnaire. Thus, the data were subject to recall and social desirability biases. Moreover, categorizing drug use into former and current categories does not account for the duration and severity of usage. However, the data collected in the 2017–2018 cycle did not include data that facilitated the categorization of users based on duration and severity.

The present study offers valuable insights, but several limitations should be acknowledged. The cross-sectional nature of NHANES prevents causal inference, and reliance on self-reported drug use introduces the risk of recall error, social desirability bias, and potential underreporting due to stigma. Exposure misclassification may therefore have influenced the observed associations. Selection bias and possible mortality bias may also be relevant, as individuals with more severe substance use or poorer oral health may have been underrepresented. Furthermore, residual confounding cannot be excluded, as data on important factors such as tobacco and alcohol consumption, diet, fluoride exposure, oral hygiene, and access to or insurance for dental care were unavailable. Moreover, categorizing drug use into former and current categories does not account for the duration and severity of usage. However, the data collected in the 2017–2018 cycle did not include data that facilitated the categorization of users based on duration and severity.

Finally, multiple comparisons were performed, raising the possibility of type I error.

The findings from this study underscore important implications for both dental professionals and policymakers. For clinicians, awareness of the heightened risk of caries among individuals with a history of illicit drug use highlights the need for early detection, preventive counseling, and tailored treatment strategies that account for xerostomia, bruxism, and dental avoidance behaviors. For policymakers, the results emphasize the importance of integrating oral health services within broader substance use and mental health programs, reducing barriers to dental care, and expanding coverage for preventive interventions. By addressing the intersection of drug use, mental health, and oral health at both the clinical and policy levels, more effective and equitable strategies can be developed to reduce the burden of dental disease in this vulnerable population.

## CONCLUSION

Significant associations were observed between illicit drug use and the severity of caries, as measured using the DMFT index, in the present study. Different association patterns were observed for different drugs, indicating the different effects of drugs on the development of caries based on their chemical composition, method of administration, and mechanism of action. Illicit drug users present with several biological, psychological, social, and mental factors that must be treated during and after the cessation of addiction. Oral health providers and policymakers should screen for signs of substance use, facilitate adequate management of the consequences of dental diseases, and refer patients to address psychological, social, and mental challenges, if required.

## ACKNOWLEDGMENTS

The project was funded by KAU Endowment (WAQF) at King Abdulaziz University, Jeddah, Saudi Arabia. The authors therefore gratefully acknowledge WAQF and the Deanship of Scientific Research (DSR) for technical and financial support.
